# A Spiropyran-Based Hydrogel Composite for Wearable Detectors to Monitor Visible Light Intensity to Prevent Myopia

**DOI:** 10.1021/acsami.5c00250

**Published:** 2025-01-27

**Authors:** Jiaxin Zhang, Mengxia Lu, Xin Cai, Peter Müller-Buschbaum, Qi Zhong

**Affiliations:** † Key Laboratory of Advanced Textile Materials & Manufacturing Technology, Ministry of Education, Zhejiang Sci-Tech University, 928 Second Avenue, 310018 Hangzhou, China; ‡ Key Laboratory of Silk Culture Heritage and Products Design Digital Technology, Ministry of Culture and Tourism, School of Fashion Design and Engineering, Zhejiang Sci-Tech University, 310018 Hangzhou, China; § Keyi College of Zhejiang Sci-Tech University, 58 Kangyang Road, 312369 Shaoxing, China; ∥ TUM School of Natural Sciences, Department of Physics, Chair for Functional Materials, Technical University of Munich, James-Franck-Str. 1, 85748 Garching, Germany

**Keywords:** spiropyran, hydrogel composites, visible light detector, reversible photochromism, myopia prevention

## Abstract

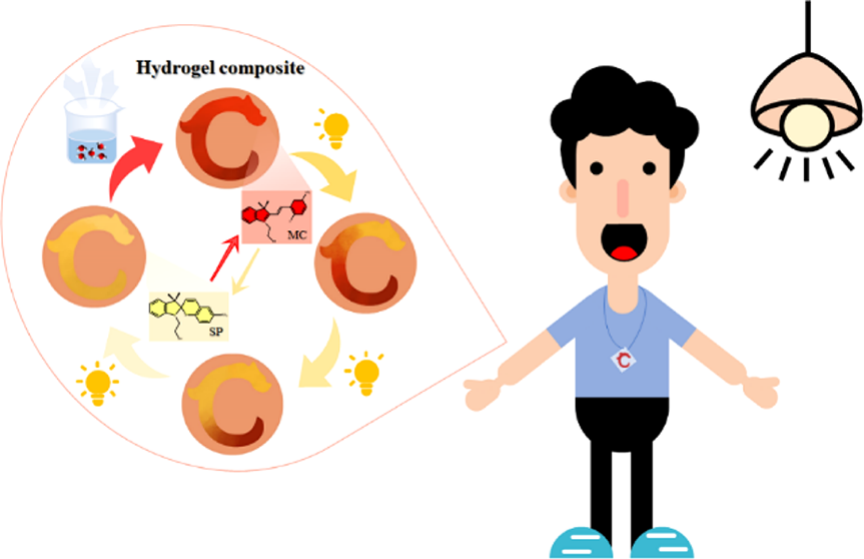

A wearable detector to monitor visible light intensity is realized by the restrained photochromism of a hydrogel composite containing light-responsive spiropyran with hydroxyl groups (SPOH). When exposed to visible light, the SPOH experiences a ring-opening to a ring-closed transition accompanied by discoloration from red to yellow. Unlike in the solution, the photochromism/discoloration rate is strongly correlated to the cross-linking points. By reducing the amount of cross-linker from 40 to 5 mg, the photochromism rate of SPOH is 300% faster. Inspired by the Chinese Jade Loong from Hongshan, the hydrogel composite is shaped into a Loong to monitor the light intensity. By increasing the amount of cross-linker in the head, body, and tail, the photochromism/discoloration rate sequentially turns slower from one region to the other. Higher light intensity is required to realize the discoloration in the hydrogel composite containing a larger amount of the cross-linker. Because the initial colors are identical, the light intensity can be easily traced by checking the discoloration of these pieces containing different amounts of cross-linker. Based on this unique and reversible photochromic capability, the present hydrogel composite can be used for monitoring the visible light intensity to prevent myopia, especially for children and students.

## Introduction

Comfortable light conditions are an important issue in our daily environment.^1,2^ Insufficient or extensive visible light intensity may cause visual fatigue, dryness, and tearing of the eye, especially when reading under such conditions. Moreover, it can even affect vision and induce myopia in serious cases, thus affecting the reading and learning efficiency of children or students.^3−8^ For this reason, detectors to monitor the visible light intensity are highly desired to avoid such eye damage. Over the past few decades, a variety of detectors have emerged to monitor the visible light intensity in indoor environments.^9−12^ Currently, commercially available illuminometers can measure the visible light intensity accurately. However, they are not only expensive but also require expertise to measure the intensity, which renders them unsuitable for typical private households.^13,14^ There are also detectors that utilize charge-coupled devices (CCDs) to harvest light to obtain visible light intensity with high accuracy. However, as specialized equipment, technicians are required to analyze and process the data, which again makes such equipment unsuitable for children and students.^15,16^ Therefore, detectors that are easy to use, cheap, and efficient to monitor the visible light intensity are highly desired if indoor light intensity measurements are to be made available to private households.

Recently, stimulus-responsive materials have been broadly used in various fields due to their unique capability to respond to stimulation from an external environment.^17,18^ Among these stimuli, light has the advantages of controllable wavelength and energy, in combination with being noninvasive and nondestructive. For this reason, photochromic materials that can be excited via light become promising candidates to monitor visible light intensity. The present photochromic materials can be divided into inorganic and organic ones, which are broadly used for UV detection, anticounterfeiting, and smart textiles.^19−23^ In general, inorganic photochromic materials have the characteristics of being antioxidant, having good thermal stability, and having well-behaved mechanical properties. However, their slow response and single optical property limit their applications.^24^ For instance, WO_3_ undergoes discoloration by generating electrons upon exposure to light, which further induces absorption in the long-wavelength region. However, due to the severe electron–hole recombination, the photochromic efficiency is low.^25^ In addition, TiO_2_ nanoparticles have also been extensively reported as a photochromic material, but their high photochromic response can only be observed when the nanoparticles are dispersed in a liquid state containing a strong photogenerated hole scavenger.^26^ On the other hand, organic photochromic materials present a number of advantages, such as high photosensitivity, excellent fatigue resistance, and recyclability.^27^ Among them, spiropyran (SP) and its derivatives can switch from a ring-open state (colored monomer) to a ring-close state (colorless spiral ring) when exposed to visible light.^28−33^ In addition, this transition is reversible, indicating that the ring will be back to the open state upon exposure to UV radiation. So far, investigations about spiropyran-based photochromic materials have mainly focused on UV-induced photochromism and its corresponding applications, such as UV detection, anticounterfeiting, and information encryption. However, to the best of our knowledge, most of the reported works are focused on utilizing their positive photochromic properties, i.e., ring-closed to ring-open transition upon UV irradiation to achieve anticounterfeiting or UV-indicating applications, and then utilizing visible light for recycling or fatigue resistance. Unlike the reported work, our investigation is mainly based on the negative photochromic property of spiropyran.^34^ It can switch from a colored to colorless state after visible light illumination. Because the transition of spiropyran under visible light illumination is strongly correlated to the visible light intensity, it can be used to monitor the intensity of visible light for the prevention of myopia. Although the spiropyran molecules can switch from a ring-open to ring-close state after applying the heat treatment, the spiropyran molecules can also switch from a colored (ring-open) to colorless (ring-close) state after visible light illumination by the negative photochromic property. Therefore, the main novelty of our present work is to design a visible light detector based on the photochromism of spiropyran from a ring-open to ring-closed state upon exposure to visible light. By simply tracing the discoloration of the hydrogel composite in seconds, one can monitor the visible light intensity in an indoor environment. This visible light detector can efficiently prevent myopia in students and children. Therefore, it is feasible to design detectors to monitor visible light intensity via the application of SP-based organic photochromic compounds. However, SP is usually in powder form under ambient conditions, which presents a slow photochromism rate and weak response to light. In addition, the powder form is difficult to handle in our daily lives. Therefore, a suitable carrier is highly desired for the SP-based photochromic materials to achieve efficient photochromism and wearable capability.^35^ Hydrogels are cross-linked polymers with a three-dimensional structure. Due to their excellent flexibility and stretchability,^36,37^ they are widely used as artificial skin,^38−40^ wearable electronics,^41^ sensors,^42−44^ and self-healing materials.^45^ For this reason, hydrogels are suitable as the carrier for SP. Although the cross-linking points in hydrogels provide good mechanical properties,^46−49^ they also significantly reduce the space for chain arrangement. Because the photochromism of SP is strongly related to the ring open–close processes, the lack of space in hydrogels is not beneficial for visible light-induced photochromism.^50^ Therefore, simultaneously realizing good mechanical properties and excellent photochromic behavior is still a challenge in the hydrogel composites containing SP.

Based on the above discussion, the present investigation is mainly focused on optimizing the cross-linking extent in the hydrogel composite containing SP to achieve visible light intensity-correlated photochromic behavior. By introducing additional hydroxyl groups to SP, the obtained SPOH presents better compatibility with hydrogels. Inspired by the ancient Chinese Jade Loong from Hongshan, the obtained hydrogel composite is shaped into a Loong.^51^ By increasing the amount of cross-linker (*N*,*N*′-methylene-bis-acrylamide, MBA) from head to tail in the Loong-shaped hydrogel composite, the photochromism/discoloration rates sequentially turn slower in these three pieces, although the initial colors are identical (red color). Therefore, the visible light intensity can be monitored by simply tracing the extent of the discoloration in different pieces. Based on the reversibility of SPOH, the color can recover to its original state by simply mounting the hydrogel composite in warm water thermostated at 50 °C. Conventional photochromic materials often rely on UV irradiation to trigger color change.^52,53^ Thus, they are mainly used for UV detectors or sensors. However, in our present investigation, the hydrogel composites are designed to respond to visible light, which is used to effectively monitor the visible light intensity in the environment for the prevention of myopia based on photochromism. In addition, the hydrogel composites have significant advantages in terms of flexibility and wearability, which can be used to prepare smart clothing and wearable sensors. Such characteristics make the hydrogel composites more portable and practical for daily use, whereas traditional photochromic materials are commonly somehow restrained in their applications in daily life due to their lack of flexibility and wearability. Although Li et al. synthesized a coordination polymer with photochromic properties based on viologen (*N*,*N*′-disubstituted 4, 4′-bipyridine) derivatives for test papers sensitive to UV and visible light,^11^ the visualized monitoring of visible light intensity is still not possible because the discoloration is not prominent under visible light. Therefore, to the best of our knowledge, the visualized visible light detector based on the ring-open to ring-close of spiropyran is first reported in our present work. Based on the advantages of facile preparation, light intensity sensitivity, excellent flexibility, and good recyclability, the present hydrogel composite is a promising candidate for a wearable detector to monitor the visible light intensity.

## Experimental Section

### Materials

The monomers sodium alginate (SA, AR), 2-methyl-2-propenoic acid-2-(2-methoxyethoxy) ethyl ester (MEO_2_MA, purity of 95%), poly(ethylene glycol) methyl ether methacrylate (OEGMA_300_, purity of 95%), and calcium chloride (CaCl_2_, AR) were purchased from Sigma-Aldrich. Ammonium persulfate (APS, purity of 99.99%) and *N*,*N*,*N*′,*N*′-tetramethylethylenediamine (TEMED, AR) were purchased from Aladdin. *N*,*N*′-methylene-bis-acrylamide (MBA, purity of 99%), 2,3,3-trimethylindolenine (purity of 99%), 5-nitrosalicylaldehyde (purity of 98%), 2-bromoethanol (purity of 99%), *n*-hexane (purity of 97%), and acetonitrile (purity of 99%) were bought from Macklin. Potassium hydroxide (KOH, AR) and ethanol (EtOH, AR) were obtained from Gaojing Chemical.

### Preparation of SPOH

SPOH was synthesized according to the literature.^61^ Before the synthesis, 2,3,3-trimethylindolenine (12.6 mmol, 2.0 g) and 2-bromoethanol (15.1 mmol, 1.7 g) were refluxed in acetonitrile (25 mL) under a N_2_ atmosphere for 24 h. After the reaction at 80 °C for 24 h, the mixture was washed with hexane (25 mL) three times and concentrated in vacuum. Then, an aqueous KOH solution (40 mL, 30 mg mL^–1^) was added to the flask containing the obtained purplish-red solid (2.8 g). After the reaction at 25 °C for 30 min, the product was extracted with ether (20 mL) three times and washed with deionized water (30 mL) three times. After being concentrated in a vacuum, the obtained yellow oil (7.4 mmol, 71.5 mg) was dissolved in a mixed solution containing ethanol (15 mL) and 5-nitrosalicylaldehyde (11.1 mmol, 1.9 g). After refluxing in N_2_ atmosphere for 5 h, the flask was cooled to room temperature. A purple filter cake was obtained after filtering. Further washing with ethanol and treatment with vacuum drying, the final product SPOH was obtained. The synthesis process of the SPOH is shown in Figure S1.

### Preparation of the SPOH-Based Hydrogel Composite

Scheme S1 presents a schematic diagram of the design and principle of the hydrogel detector. SPOH was used to prepare the P(MEO_2_MA-*co*-OEGMA_300_)/SPOH hydrogel composite. Briefly, the polymerization process was as follows: SA (0.025 g, 0.115 mmol), MEO_2_MA (0.924 mL, 10 mmol), MBA (5 mg, 0.026 mmol), OEGMA_300_ (1.428 mL, 10 mmol), and SPOH (10 mg) were sequentially added to deionized water (10 mL). After treatment with ultrasound for 30 min and stirring for 120 min, the obtained solution was degassed with N_2_ for 30 min to remove possible oxygen. Then, APS (12 mg, 52.8 mmol) and TEMED (12 μL, 0.08 mmol) were added under N_2_ protection. After the mixed solution was cured at 30 °C for 12 h, the hydrogel composite was immersed in a CaCl_2_ solution (40 mL, 10 mg mL^–1^) for 1 min to harden the surface. Finally, the hydrogel composite was placed into deionized water for 10 min to remove the residual CaCl_2_ solution.

To address the influence of the cross-linker on the photochromism, the amount of MBA was increased to 20 mg (0.104 mmol) and 40 mg (0.208 mmol). The preparation protocols for the hydrogel composite were identical.

### Preparation of Visible Light Detector with Loong Shape

The Loong-shape hydrogel composite was separated into three pieces as head, body, and tail. The amounts of cross-linker MBA were sequentially increased from 5 mg (head) to 20 mg (body) and 40 mg (tail) and finally sealed with plastic foil (Deli 14888, China). Thus, although the initial colors of these three pieces are identical to red, the photochromism/discoloration rates turn slower in sequence from head to tail.

### ^1^H NMR Spectroscopy

The ^1^H NMR spectra of SPOH were measured by a nuclear magnetic resonance spectrometer (Avance AV 400 MHz, Bruker, Switzerland). Tetramethylsilane (TMS) and CDCl_3_ were used as the internal standard and solvent, respectively.

### ATR-FTIR Spectroscopy

The functional groups in SPOH powder, pure P(MEO_2_MA-*co*-OEGMA_300_) hydrogels, and the P(MEO_2_MA-*co*-OEGMA_300_)/SPOH hydrogel composite were probed by attenuated total reflection Fourier-transform infrared (ATR-FTIR) spectroscopy (Vertex 70 spectrometer, Bruker, USA). The wavenumbers ranged from 600 to 4000 cm^–1^. The corresponding scan number and resolution were 64 and 4 cm^–1^, respectively.

### FE-SEM Measurements

The surface morphology and internal structure of the hydrogel composites were probed by field emission scanning electron microscopy (FE-SEM) (ULTRA 55, Carl Zeiss SMT Pte Ltd., Germany). The corresponding working voltage and distance were 3 kV and 8 mm, respectively. Before the measurements, the pure and hydrogel composites were sputtered with platinum at 20 mA for 110 s by an automatic fine coater (JFC-1600, JEOL, Japan). The operating voltage was switched to 10 kV, and the working distance remained unchanged during the EDS measurements.

### UV–Vis Spectroscopy

The UV–vis absorbance of the P(MEO_2_MA-*co*-OEGMA_300_)/SPOH hydrogel composite was measured by a UV–vis spectrophotometer equipped with an integrating sphere (UV-2600, PerkinElmer, USA). The measured wavelength ranged from 400 to 700 nm.

### Measurements for Visible Light Detection

A xenon lamp (HDL-II, Bo Bei Lighting Electric Appliance Factory, China) was used to mimic visible light in the measurements. The light intensity of the xenon lamp was determined with a laser power meter (LP-3B, Beijing Wuke Optoelectronics Technology Co., Ltd., China). By adjusting the power of the xenon lamp, the obtained light intensity was varied between 0 and 640 W m^–2^ (0–600 Lux) when the distance between the visible light detector and the light source was fixed at 10 cm. The *K*/*S* values of the hydrogel composite before and after illumination were measured with a color measurement and matching instrument (DC 600, Datacolor Company, USA). Therefore, the detection capability for the visible light intensity was evaluated by the change in *K*/*S* values in each part of the hydrogel composite after exposure to different light intensities from xenon illumination.

## Results and Discussion

### Structure and Morphology of Hydrogel Composite

Figure S2 presents ^1^H NMR spectroscopy data to confirm the successful synthesis of SPOH. Signals a and b are ascribed to the two methyl groups (−CH_3_), whereas signals c and d are assigned to the methylene groups attached to the hydroxyl groups. Signals e and h are attributed to the hydrogen peaks of the double-bonded carbons (−CH=CH−), and signals f, g, i, and k are from the hydrogen peaks on the benzene ring. Due to its low activity, the signal from the hydroxyl hydrogens is not prominent. ATR-FTIR is applied to investigate the functional groups in the P(MEO_2_MA-*co*-OEGMA_300_)/SPOH hydrogel composite. In the ATR-FTIR spectra of SPOH (black curve in Figure S3), the characteristic peaks related to the 1,2,4-substitute aromatic ring and Ar–C and −OH groups are visible at 830, 1250, and 3340 cm^–1^, respectively. In the case of the pure P(MEO_2_MA-*co*-OEGMA_300_) hydrogel (red curve in Figure S3), the absorption peaks related to −C–O–C– and −C=C– groups are located at 1091 and 1656 cm^–1^, respectively. For the P(MEO_2_MA-*co*-OEGMA_300_)/SPOH hydrogel composite (blue curve in Figure S3), all characteristic peaks mentioned above are observed. Therefore, it can be concluded that SPOH is successfully introduced into the hydrogel composite.

**Figure 1:**
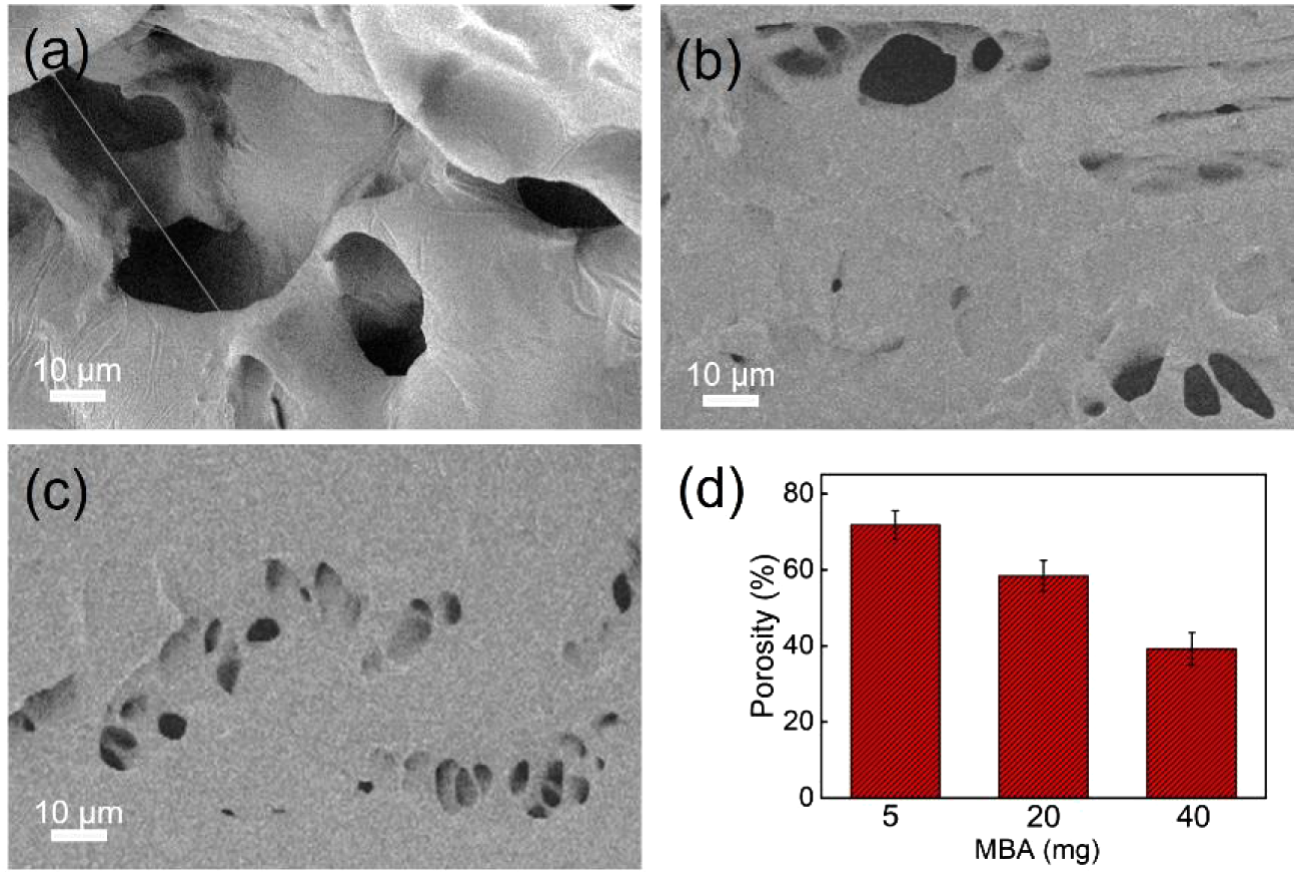
SEM images of hydrogel composites with different amounts of MBA: (a) 5 mg, (b) 20 mg, and (c) 40 mg. (d) Porosity in hydrogel composites with different amounts of MBA.

The internal structure of the hydrogel composites with different amounts of MBA is probed by FE-SEM. When the amount of MBA is only 5 mg, larger pore sizes (diameter of 20 ± 5 mm) and lower density are observed in the cross-section (Figure 1a). The presented structure is related to the fewer cross-linking points due to the lower amount of MBA. After the amount of MBA was increased to 20 and 40 mg, a smaller pore size and higher densities are visible in Figure 1b,c, respectively. In addition, by comparing the porosity of hydrogel composites containing different amounts of MBA (Figure 1d), the porosity of the hydrogel composites gradually decreases with the amount of MBA. Thus, this again confirms the influence of the MBA amount on the porous structure in the hydrogel composites. The tendency in the hydrogel morphology indicates that the amount of cross-linker MBA in the hydrogels is strongly correlated to the network structure. An increased amount of MBA induces the denser packing of the polymer chains. It limits the free space for the transition from the ring-open to ring-close state in SPOH, which further prevents its photochromism. Table S1 summarizes the element contents in the hydrogel composites containing different amounts of MBA. An increasing tendency is observed when element N in these three hydrogel composites is compared. Because only MBA possesses element N, the finding confirms that there is more cross-linker MBA in the hydrogel composite when the added amount is increased. The desired application of hydrogel composites is the prevention of myopia. For this reason, the materials used for preparation should be cheap and accessible. MBA is a common and readily available cross-linker. By simply adjusting the amount of MBA in the hydrogel composites, the cross-linking density, as well as the photochromic behavior, can be regulated.

### Photochromic Behavior of Hydrogel Composites

**Figure 2:**
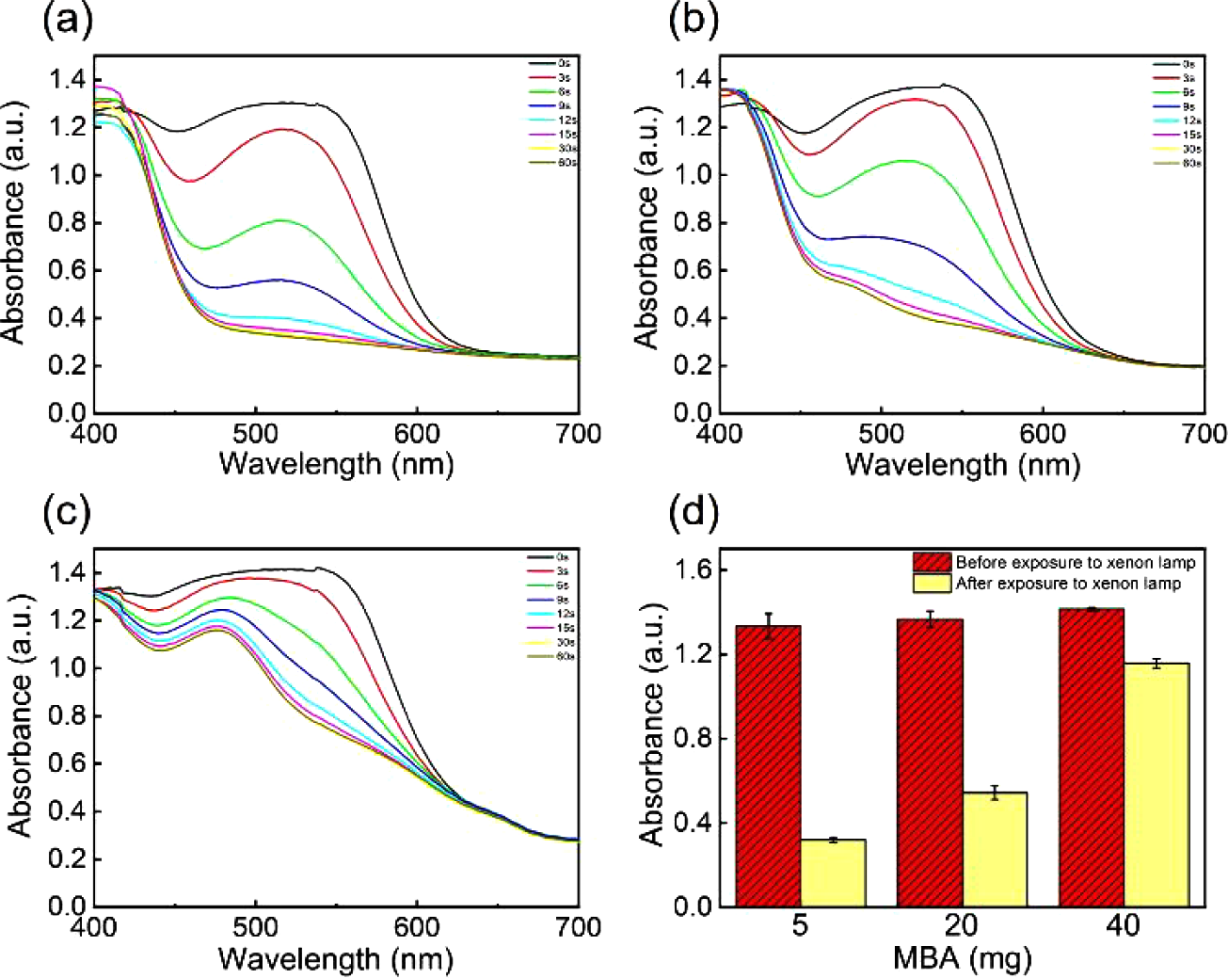
Photochromic behavior of hydrogel composites with the different amounts of MBA (a: 5 mg, b: 20 mg; and c: 40 mg). (d) The absorbance value of hydrogel composites containing different amounts of MBA (5, 20, and 40 mg) at the maximum absorption peak before and after exposure to the xenon light. The light intensity applied is fixed as 637 W m^–2^.

The photochromic behaviors of the P(MEO_2_MA-*co*-OEGMA_300_)/SPOH hydrogel composites are probed by a UV–vis spectrophotometer (Figure 2a–c). When the amount of MBA in the hydrogel composite is 5 mg, the absorbance value at the maximum absorption peak (550 nm) before illumination by the xenon lamp is 1.3. After illumination, the maximum absorption peak is shifted to 490 nm, and the absorbance value is reduced to 0.3. This prominent shift of the peak position and reduction of the absorbance is caused by the discoloration from red to yellow via the ring-open to ring-close transition of SPOH. Due to the limited amount of MBA, the cross-linking points in the hydrogel composite are rare. Thus, the SPOH component provides sufficient space to realize the transition. When the MBA amount is increased to 20 mg, the initial absorbance value before illumination is still 1.3, but after illumination, the value is decreased to only 0.6 instead of 0.3. Thus, the change is smaller when the MBA amount is increased. The less prominent change is caused by the increased number of cross-linking points in the network. It significantly shrinks the available space for the transition in SPOH. Further increasing the MBA amount to 40 mg, the change of absorbance value is further reduced to 0.2 (Figure 2d). By comparison of the photochromic behavior of hydrogel composites with different amounts of MBA, the excitation energy can be obtained. The hydrogel composite with 5 mg of MBA can be discolored in 10 s at a light intensity of 637 W m^–2^. Thus, the excitation energy can be calculated as 6.4 × 10^3^ J m^–2^. Similarly, the excitation energy of the hydrogel composite with 20 and 40 mg of MBA can be calculated as 9.5 × 10^3^ and 12.7 × 10^3^ J m^–2^, respectively. Therefore, the excitation energy increases with the extent of cross-linking. This finding again confirms that the transition capability of SPOH from the ring-open to ring-closed state is strongly correlated with the free space in the hydrogels. Besides the amount of MBA, the other factors, such as polarity^54^ and hydrogen bonding^55^ in the hydrogels, will also play a role in the photochromism of spiropyran.

To improve the mechanical properties, the hydrogel composites are immersed into a CaCl_2_ aqueous solution, which triggers the formation of physical cross-linking in the hydrogels. Combined with the chemical cross-linking, the thereby formed interpenetrating network (IPN) structure can profoundly enhance the mechanical properties of the hydrogels, which is favorable for the daily wear of the constructed detector. However, the additional physical cross-linking points may also affect the transition capability of SPOH. For this reason, the influence of immersion time in the CaCl_2_ aqueous solution on the photochromic behavior is investigated as well. According to the Kubelka–Munk staining depth equation, the absorption coefficient K and the scattering coefficient S of the measured sample have a certain functional relationship with the colored extent of the sample. Therefore, the *K*/*S* value was used to describe the color extent of the sample. As shown in Figure S4, the *K*/*S* values before and after exposure to the xenon lamp remain unchanged when changing the immersion time. This behavior illustrates that the formation of physical cross-links in the hydrogels does not influence the SPOH transition. The possible explanation might be related to the location of the physical cross-linking points. After immersion in a CaCl_2_ aqueous solution, the Ca^2+^ ions can immediately replace the Na^+^ ions in the hydrogels and form a physical cross-linking shell on the outer part of the hydrogels. However, this shell hinders the diffusion of Ca^2+^ ions into the interior part of the hydrogels. Because the maximum applied immersion time is only 4 min, no IPN structure is formed in the interior part of the hydrogels. Thus, the transition capability of SPOH is not influenced by the additional physical cross-links.

### Photochromism and Recovery Behaviors of the Hydrogel Composite

**Figure 3:**
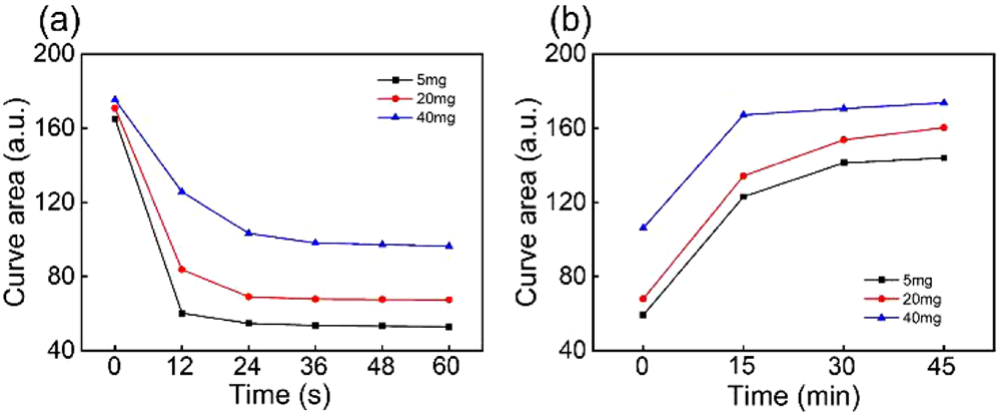
Comparison of curve areas of hydrogel composites with different amounts of MBA (a) during visible light illumination and (b) recovery in hot water. The light intensity applied is fixed as 637 W m^–2^. Solid lines are guides for the eye.

To further address the optical absorption performance of P(MEO_2_MA-*co*-OEGMA_300_)/SPOH hydrogel composites, the area of the maximum absorption peak in the UV–vis absorption spectrum from 466 to 600 nm in Figure 2a–c is integrated and denoted here as the curve area. It can be used to characterize the correlation between the maximum absorption peak and the amount of MBA. Figure 3a shows the temporal changes in the curve area after illumination for up to 60 s. The initial values for the curve area are almost identical in these three samples (165, 170, and 175), indicating that the MBA amount does not influence the initial color of the hydrogel composite. When the amount of MBA is only 5 mg, a fast and abrupt decrease can be observed (black dots). After only 12 s of illumination, the curve area drops to 60. After that, it almost remains unchanged. When the MBA amount is increased to 20 (red dots) and 40 mg (blue dots), the decrease is less prominent. After 60 s, the final values of the curve area gradually increase from 53 (5 mg of MBA) to 72 (20 mg of MBA) and 96 (40 mg of MBA). In addition, a longer time is required to reach the equilibrium state. The transition rates from the ring-open to ring-close state are 100% (20 mg of MBA) and 300% (40 mg of MBA) slower than that containing 5 mg of MBA. Thus, it can be concluded that not only the photochromic rate of SPOH but also the final state is strongly related to the available space in the hydrogels.

Besides the cross-linking density, the correlation between the discoloration and incident light intensity in hydrogel composites with different amounts of MBA is also studied. As shown in Figure S5, when the amount of MBA in the hydrogel composite is only 5 mg, a light intensity as low as 200 W m^–2^ can induce a pronounced reduction of the *K*/*S* ratio from 28.0 to 20.8 after illumination for 10 s. In contrast, the hydrogel composites with a higher extent of cross-linking only present a minor reduction of the *K*/*S* value, indicating that there is almost no color change in the same scenario. This means that the low incident light intensity is not sufficient for the excitation energy. Further increasing the light intensity to 400 W m^–2^, the *K*/*S* value significantly drops to 10.1 in the hydrogel composites with 5 mg of MBA after illumination for 10 s. Simultaneously, the *K*/*S* values of the hydrogel composite with 20 and 40 mg of MBA also decrease to 17.4 and 25.8, respectively. Further increasing the light intensity to 600 W m^–2^, both the *K*/*S* values of the hydrogel composites with 5 and 20 mg of MBA drop to 9.1, while the *K*/*S* value of the one with 40 mg of MBA reduces to 18.8 as well. Thus, the incident light intensity is strongly correlated to discoloration due to the excitation energy of SPOH.

Because the photochromism of SPOH is reversible, the recovery of SPOH from the ring-closed to ring-open state is also investigated. After placing in warm water thermostated at 50 °C, all three composites present a gradual increase of the curve area with time (Figure 3b), illustrating that their photochromic behavior is reversible. However, the composites cannot be fully recovered to their initial state. The possible reason is oxidative degradation due to irreversible changes in the molecular structure under environmental factors such as light and oxygen. Specifically, it is caused by a photooxidation process, which was previously reported by Baillet et al.^56−58^ The color of the composite can be gradually recovered from yellow to red (Figure S6). It should be noted that unlike the transition from the ring-open to ring-close state, the recovery to the ring-open state takes a much longer time (30–40 min). The possible reason might be that the rearrangement of polymer chains to realize the ring-open state requires a longer time. Photochromism is a photochemical phenomenon, indicating that the process immediately takes place when the sample is exposed to visible light illumination. However, the recovery of the hydrogel composite to its original color in hot water is a thermochemical process. For this reason, the spiropyran molecules must absorb a sufficient amount of energy to overcome the activation energy barrier and initiate a thermal reaction. Because the photochromism of spiropyran is a reversible process, we assume that the recovery energy is the same as the excitation energy. By comparing the photochromic behaviors of hydrogel composites with different amounts of MBA, the required excitation energy is related to the amount of MBA. The excitation energy for the hydrogel composite with 5, 20, and 40 mg of MBA can be calculated as 6.4 × 10^3^, 9.5 × 10^3^, and 12.7 × 10^3^ J m^–2^, respectively. The recovery energy is on the same scale as the excitation energy. Because the hydrogel composite possesses a huge number of water molecules, its larger specific heat capacity induces a slower heating rate of the hydrogel composite containing spiropyran to reach sufficient temperature and absorb enough energy to overcome the energy barrier. For this reason, the recovery process takes a much longer time than that in photochromism.

In addition, the different recovery temperatures affect the recovery process as well. To address this point, the correlation between the recovery temperature and the recovery time is investigated in the hydrogel composite with 5 mg of MBA. The recovery of the hydrogel composite almost shows a linear behavior when the water bath temperature for recovery is 40 °C (black curve in Figure S7). In addition, the final *K*/*S* value reaches only 22.4, which is much smaller than the initial value (28.0). This means that the hydrogel composite cannot fully recover to its initial state at such a low temperature. In contrast, when the recovery temperature is increased to 50 °C (red curve in Figure S7), the recovery is much faster. After only 40 min, the *K*/*S* value returns to 25.6. After 60 min, the final *K*/*S* value reached is 27.1, which is very close to that of the initial state. Further increasing the recovery temperature to 60 °C (green curve in Figure S7), the whole recovery process becomes even faster. The final *K*/*S* value reached is 27.9, indicating that the hydrogel composite almost recovers to its initial state. Therefore, it can be concluded that the recovery temperature is strongly related to the recovery process, which might be related to the lower excitation energy at a higher temperature.

### Schematics for Photochromic Behavior of the Hydrogel Composite

**Figure 4:**
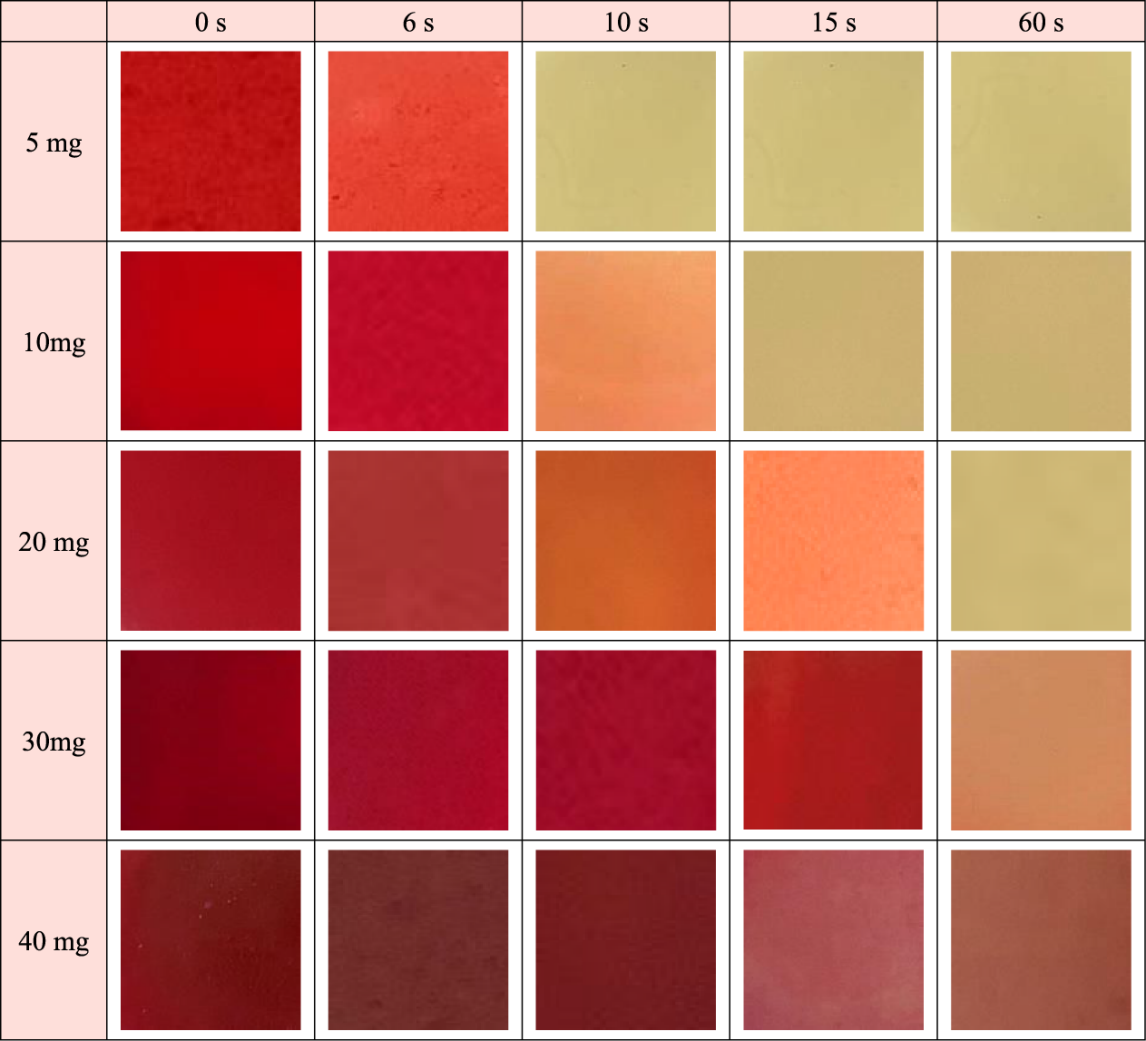
Temporal discoloration in the hydrogel composites containing different amounts of MBA (5, 10, 20, 30, and 40 mg) after illumination for 0, 6, 10, 15, and 60 s. The light intensity applied is fixed as 637 W m^–2^.

To address the photochromic behavior of the hydrogel composites as well as the influence of cross-linking extent on the photochromism, the temporal discoloration of the hydrogel composites containing different amounts of MBA was probed by optical microscopy. As shown in Figure 4, the hydrogel composite containing 5 mg of MBA can rapidly switch from red to yellow color and reach an equilibrium after illumination for only 15 s. Such behavior indicates that the lower extent of cross-linking induces more available space in the hydrogels, which is favorable for the transition from the ring-open to ring-close state. When the amount of MBA is 20 mg, a longer time is required for the discoloration to reach a yellow color. Further increasing the amount of MBA to 40 mg, even after illumination for 60 s, the hydrogel composite presents only a minor change of color to light red. To clarify the correlation between the extent of cross-linking and the photochromism of the hydrogel composites, the discoloration time for hydrogel composites containing 10 and 30 mg of MBA is also probed. As shown in Figure 4, the hydrogel composites with 5 mg of MBA can complete the photochromism from red to yellow and reach equilibrium within 10 s of illumination. When the MBA amount is increased to 10 and 20 mg, the equilibrium time is increased to 15 and even 60 s, respectively. In addition, the final colors are only a little darker than that with 5 mg of MBA. Further increasing the MBA amounts to 30 and 40 mg is also not beneficial. Although the photochromism still exists, the discoloration is not as prominent as those with less MBA. The final color is closer to red than to yellow. To address the correlation between the cross-linking extent and the photochromism rate, the response times of the hydrogel composite containing 5, 10, and 20 mg of MBA are plotted as a function of the MBA amount. The photochromism rate/response time is strongly related to the cross-linking extent (Figure S8). When the MBA amount is increased 4 times from 5 to 20 mg, the photochromism rate is 6 times slower. Thus, it can be concluded that the photochromism capability of SPOH is strongly correlated to the extent of cross-linking in the hydrogels. Because the transition from the ring-open to ring-close state requires the arrangement of the polymer chains, more space is favorable for the transition process. If there are too many cross-linking points in the hydrogel, the space for the transition is too restricted. It induces difficulties in the transition and results in a poor photochromism performance. Higher visible light intensity is required to realize the discoloration in the hydrogel composite containing a larger amount of MBA. There are only a few investigations about spiropyran-based visible light detectors. For this reason, the present work is compared with the detection sensitivity of the spiropyran-based UV detector. Wu et al. reported a colorimetric card based on a composite material of spiropyran and silicone elastomers for monitoring UV radiation. It is able to present the current intensity of UV irradiation after exposure to sunlight for 10 s. In comparison, the present hydrogel detector can also determine the visible light intensity after exposure to visible light for 10 s. Therefore, the sensitivity of the present detector is considered very good.

### Monitoring Visible Light Intensity in Hydrogel Composites

**Figure 5:**
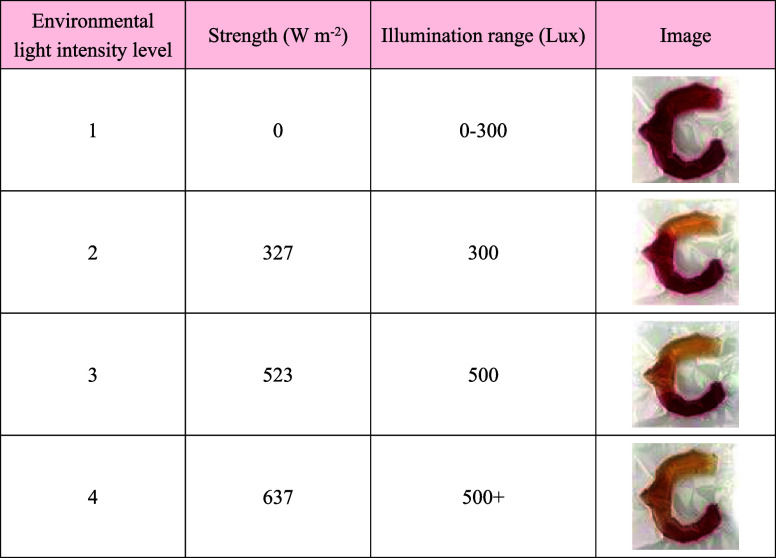
Optical image of the photochromic behavior of the Loong-shaped detector illuminated by a xenon lamp with different light intensities.

Inspired by the traditional Chinese craftsmanship of pattern expression and the elements of the ancient Chinese Jade Loong of Hongshan, a wearable detector with the shape of a Loong is designed to monitor the visible light intensity for reading. Again, the xenon lamp is used to mimic visible light in an indoor environment. To identify the visible light intensity, the amount of MBA in the detector with a Loong shape is changed from 5 (head) to 20 (body) and 40 mg (tail). The initial colors of the head, body, and tail are very close to each other (Figure 5). If the visible light intensity is lower than 327 W m^–2^ (300 Lux), then no change in the color of the detector occurs after visible light illumination for 10 s, illustrating that the environment is too dark to read well. When the light intensity reaches 327 W m^–2^, the discoloration of the head can be completed after visible light illumination for 10 s. Simultaneously, the body and tail remain unchanged. It indicates that such indoor light condition is suitable for reading without any harm to eyes.^59,60^ After recovery to its original state by mounting in warm water, the visible light intensity is increased to 503 W m^–2^ (500 Lux). After illumination for the identical time (10 s), both the head and body of the detector with Loong shape are discolored, whereas the tail still remains unchanged. This means that the visible light intensity reaches a critical value. Further increasing the light intensity will induce the environment to be too bright to read well. Such light conditions will cause dryness and tears in the eyes. When the visible light intensity reaches 637 W m^–2^ (over 500 Lux), even the tail starts to discolor after illumination for 10 s. It is a warning that the environment is too bright to read well. An immediate action to reduce the light intensity is required to protect the eyes. To address the conceptual demonstrations in real-world environments, the hydrogel composites with different amounts of MBA (5, 20, and 40 mg) are tested under natural light conditions. As shown in Figure S9a, there is almost no color change in the hydrogel composite when tested in the evening. This means that the light intensity (330 W m^–2^) is too low and not suitable for reading and writing. When switching to a more suitable natural light condition (435 W m^–2^), the head is discolored. Simultaneously, the residual parts remain unchanged (Figure S9b). This indicates that the present natural light condition is very suitable for reading and writing. When the natural light condition is switched to noon, the light intensity can reach 640 W m^–2^. Thus, both the head and body are discolored (Figure S9c). The excessively bright condition is also unsuitable for reading and writing, which should be prevented as well. The detection limit for the present hydrogel composite is 300–500 Lux, which corresponds to a suitable lighting range for reading. Therefore, based on the developed detector, the visible light intensity can be easily traced by simply checking the color of the different pieces of the hydrogel composite to prevent myopia in children and students. The temperature of the hydrogel composites with 5 mg of MBA is monitored by the IR camera before and after light illumination. As shown in Figure S10, there is almost no change in the temperature after 30 s of illumination. Thus, it can be concluded that the heat generated by illumination is so minor that it has little effect on the observed photochromic behavior.

**Figure 6:**
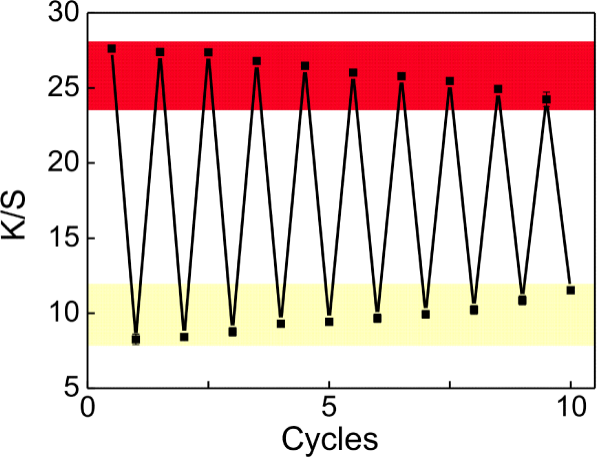
Cycling performance of the hydrogel composite containing 5 mg of MBA. The light intensity applied is fixed as 637 W m^–2^.

In addition, the recyclability of the hydrogel composite is measured. By simply immersing it in the warm water thermostated at 50 °C for 50 min, the hydrogel composite is able to recover to its initial state. To address the recyclability, *K*/*S* before and after illumination is measured for 10 cycles of illumination and recovery. Although the present hydrogel composites possess reversible photochromic properties, the oxidation process of spiropyran, inducing the photochromic behavior, worsens with time. As shown in Figure 6, even after 10 cycles of photochromism and recovery, the discoloration is still prominent from the change of *K*/*S* values, which has decreased to 65.7% of its initial value. Based on this decrease, we have extrapolated the cycle number when reaching 50% of the *K*/*S* value to be 12. Therefore, it can be concluded that the present hydrogel composite can be used at least 10 times. Because it is designed to determine visible light intensity in an indoor atmosphere, a typical use once or twice a day will be sufficient. Thus, the hydrogel composite can be used for at least 12 days before degradation. Based on the advantages of easy availability of raw materials, facile preparation, sensitivity to visible light intensity, good flexibility, and recyclability, the present hydrogel composite is designed for wearable detectors to monitor visible light intensity, which is very suitable to determine whether the light intensity is sufficient for reading in an indoor atmosphere. Hence, they can effectively prevent myopia in students and children.

**Figure 7:**
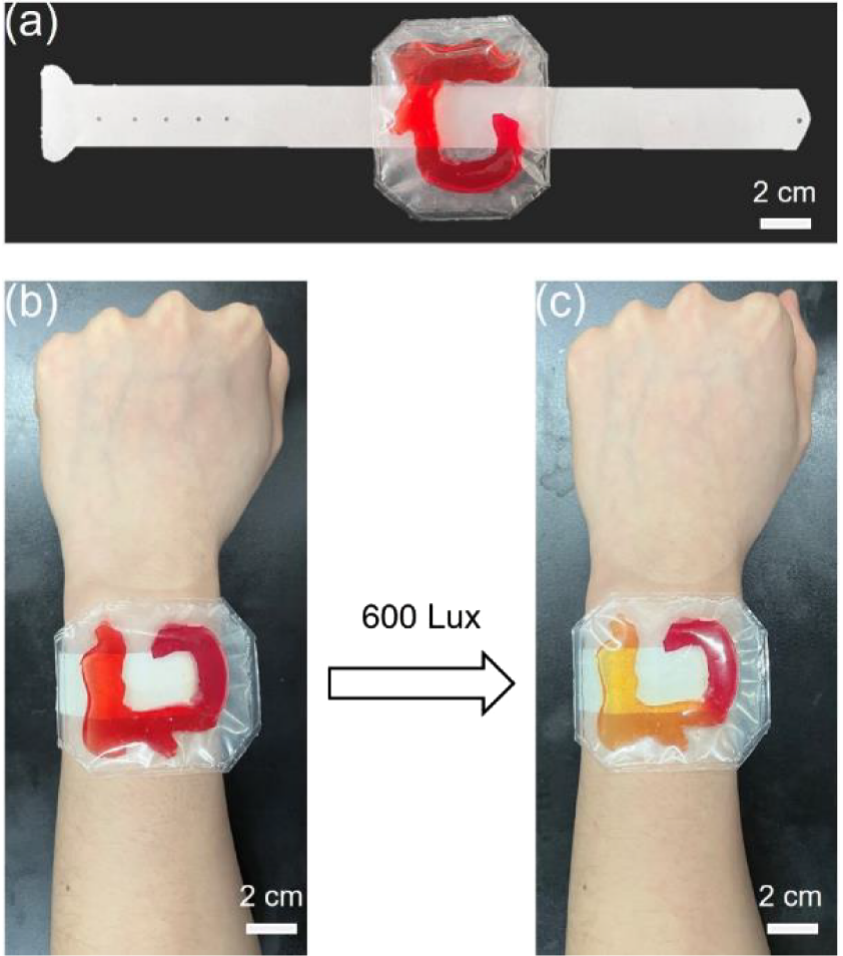
Photographs of (a) the wearable bracelet based on the hydrogel composite with different amounts of MBA (5, 20, and 40 mg) for the visible light intensity monitoring, (b) before and (c) after xenon lamp illumination with a light intensity of 600 Lux (640 W m^–2^).

Wearable sensors are prepared by vacuum encapsulation of the hydrogel composites with different amounts of MBA (5, 20, and 40 mg) by PET foils. The obtained wearable bracelet can be comfortably worn around the wrist to monitor the visible light intensity (Figure 7a). In addition, Figure 7b,c shows photographs of the wearable detector before and after illumination with high light intensity (640 W m^–2^, 600 Lux), respectively. As demonstrated, the head and body of the hydrogel composite are discolored. This indicates that it is feasible to monitor the visible light intensity by discoloration to prevent myopia

In general, discoloration takes place only when the hydrogel composite is exposed to visible light illumination with sufficient intensity. When the detector is exposed to fluctuating visible light illumination, whose maximum intensity is below 300 Lux, there is no response. When the maximum intensity of the fluctuating visible light illumination is above 300 Lux, but the minimum value is still below 500 Lux, a color change can take place. However, the response time will be longer than the illumination time with constant light intensity. When the minimum value is also above 500 Lux, the fluctuating visible light illumination does not influence the color change. The response time of the hydrogel composite is relatively fast. It varies between 10 s for the head and 20 s for the body, which is related to the extent of cross-linking of the hydrogel composite. The more MBA that is in the hydrogels, the longer the response time. Based on the proposed application, 10–20 s is fast enough for the visualized monitoring of visible light intensity in an indoor environment. The hydrogel composites are prepared by vacuum encapsulation in PET foil. Therefore, the evaporation of water from the hydrogel composites can be well prevented. In a cool and dark environment, the obtained hydrogel composites can exhibit good storage stability.

## Conclusion

A wearable detector to monitor the visible light intensity is realized based on P(MEO_2_MA-*co*-OEGMA_300_)/SPOH hydrogel composites. By varying the amount of cross-linker MBA in the hydrogels, the free space for the transition from a ring-close to ring-open state in SPOH can be adjusted. Therefore, not only the photochromic extent but also the photochromic rate of SPOH can be well controlled. Inspired by the Jade Loong of Hongshan, the hydrogel composite is used to prepare a wearable detector with a shape of the Loong to monitor the visible light intensity. By gradually increasing the amount of MBA in the head, body, and tail, the photochromic rate is reduced from part to part, although the initial state of color is identical as red. When the visible light intensity is lower than 300 Lux, there is no discoloration after illumination for 10 s. It indicates that the environment is too dark to read. When the light intensity is between 300 and 500 Lux, only the head discolors to light yellow after illumination for 10 s. It means that the light intensity is very suitable for reading, and there is no harm to the eyes. When the light intensity reaches 500 Lux or is even above, not only the head but also the body and tail discolor as well. It illustrates that the environment is too bright to read. In addition, the present visible light-induced photochromism is reversible. Based on this unique photochromic capability, the P(MEO_2_MA-*co*-OEGMA_300_)/SPOH hydrogel composites can be used for monitoring the visible light intensity to prevent myopia, especially for children and students.

## Supporting Information Available

The scheme for the schematic diagram of the design and principle of the hydrogel detector; the synthesis of SPOH; ^1^H NMR spectrum of SPOH; ATR-FTIR spectra of SPOH; hydrogels and hydrogel composite; EDS element analysis of hydrogel-based composite containing different amounts of MBA; comparison of photochromic behavior of hydrogel composites immersed in calcium chloride solution for different time; influence of light intensity to the discoloration behavior of hydrogel composites with different amounts of MBA; recovery behavior of hydrogel composites with the different amounts of MBA; the effect of different temperatures on the recovery process of the hydrogel composites; illumination time required for the hydrogel composites to reach photochromic equilibrium as a function of the MBA content; the discoloration of the hydrogel composites with different MBA under natural light conditions; the temperatures of the hydrogel composite before and after illumination for different time (PDF)
