# Supplementary material for: A Spiropyran-Based Hydrogel Composite for Wearable Detectors to Monitor Visible Light Intensity to Prevent Myopia
Source: ACS Appl Mater Interfaces. 2025 Jan 27;17(5):8445–55. doi: 10.1021/acsami.5c00250 (PMC11803555; doi:10.1021/acsami.5c00250)
Supplement: Supplementary file 1 — am5c00250_si_001.pdf [file am5c00250_si_001.pdf]

Supporting Information

Spiropyran-based Hydrogel Composite for Wearable Detector to Monitor Visible Light Intensity to Prevent Myopia

Jiaxin Zhang,[1] Mengxia Lu,[2] Xin Cai,[2,3] Peter Müller-Buschbaum[4,*] and Qi Zhong[1,4,*]*

[1]Key Laboratory of Advanced Textile Materials & Manufacturing Technology, Ministry of Education, Zhejiang Sci-Tech University, 928 Second Avenue, 310018 Hangzhou, China

[2]Key Laboratory of Silk Culture Heritage and Products Design Digital Technology, Ministry of Culture and Tourism; School of Fashion Design and Engineering, Zhejiang Sci-Tech University, 310018 Hangzhou, China.

[3]Keyi College of Zhejiang Sci-tech University, 58 Kangyang Road, 312369 Shaoxing, China

[4]Technical University of Munich, TUM School of Natural Sciences, Department of Physics, Chair for Functional Materials, James-Franck-Str. 1, 85748 Garching, Germany

*Corresponding author.

muellerb@ph.tum.de, Phone +49 89 289 12451, fax +49 89 289 12 473

qi.zhong@zstu.edu.cn, Phone +86 571 86843436 Fax +86 571 86843436

xcc516@zstu.edu.cn, Phone +86 575 82978009 Fax +86 575 82978009

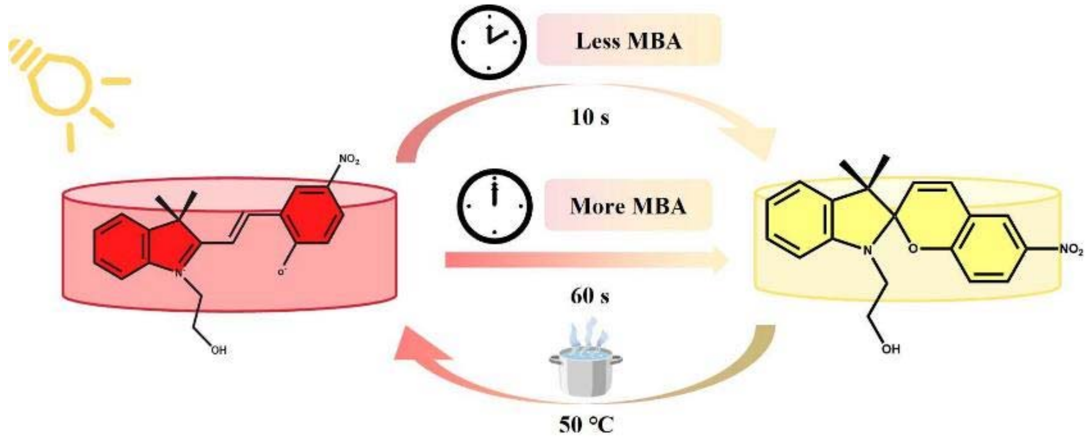

Scheme S1. Schematic diagram of the design and principle of the hydrogel detector.

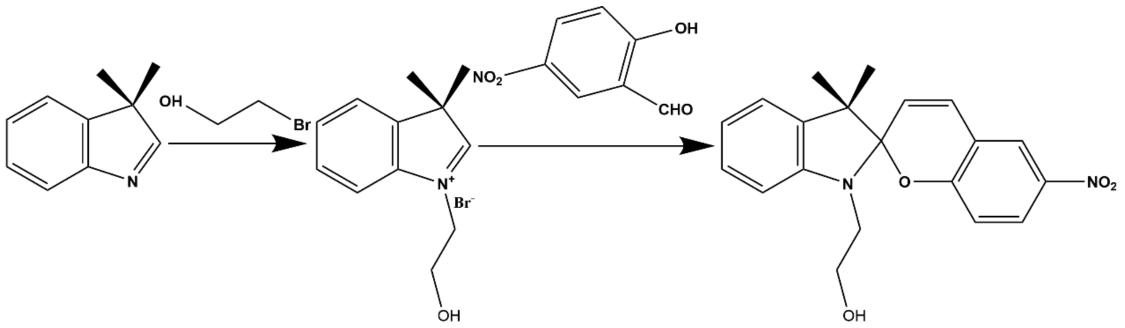

Figure S1. Scheme for the synthesis of SP containing hydroxyl groups (SPOH).

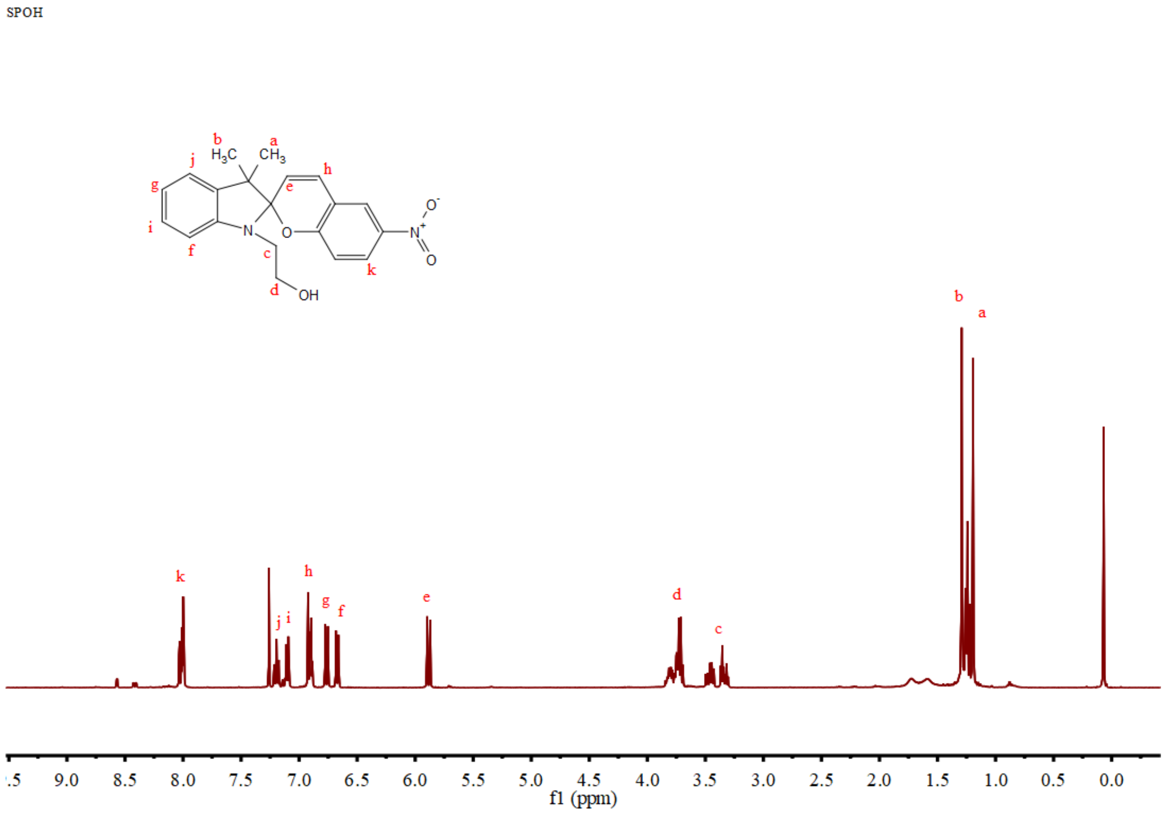

Figure S2. ^{1}H NMR spectrum of SPOH (400 MHz).

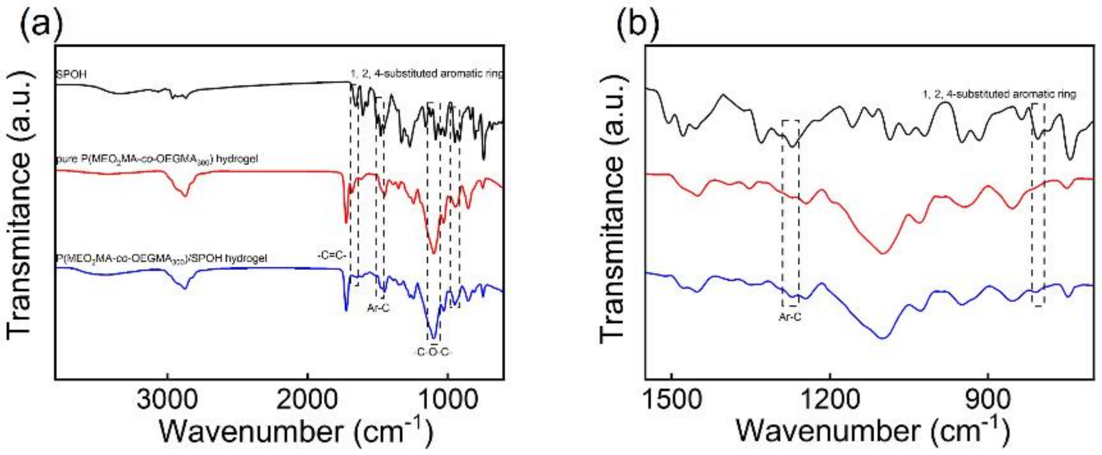

Figure S3. (a) ATR-FTIR spectra of SPOH (black), pure P(MEO_2MA-*co*-$OEGMA_{300}$) hydrogels (red), and P(MEO_2MA-*co*-$OEGMA_{300}$)/SPOH hydrogel composite (blue). (b) Zoom-in view of a specific wavenumber range.

Table S1. EDS element analysis of hydrogel composite containing different amounts of MBA: (a) 5 mg, (b) 20 mg, and (c) 40 mg.

(a)

Element	At.NO.	Mass Norm. [%]	Atom [%]
C	6	42.8	49.3 ± 0.7
N	7	9.1	9.0 ± 0.4
O	8	48.1	41.7 ± 0.7
		100	100

(b)

Element	At.NO.	Mass Norm. [%]	Atom [%]
C	6	46.6	52.9 ± 1.2
N	7	9.2	9.1 ± 0.6
O	8	44.4	38.0 ± 1.6
		100	100

(c)

Element	At.NO.	Mass Norm. [%]	Atom [%]
C	6	47.0	53.5 ± 0.3
N	7	9.5	9.3 ± 0.7
O	8	43.5	37.2 ± 0.5
		100	100

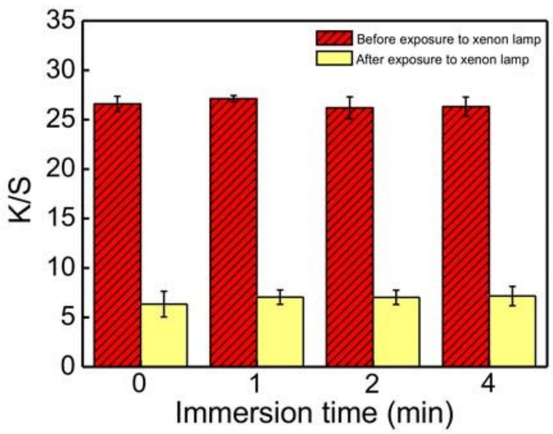

Figure S4. Comparison of photochromic behavior of hydrogel composites immersed in calcium chloride solution for different times (0, 1, 2, and 4 min). The light intensity applied is fixed as 637 W m^{-2}.

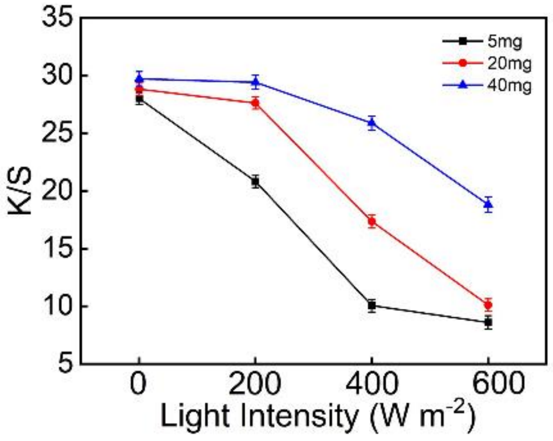

Figure S5. Influence of light intensity on the discoloration behavior of hydrogel composites with different amounts of MBA (black: 5 mg, red: 20 mg, blue: 40 mg). The illumination time is fixed as 10 s.

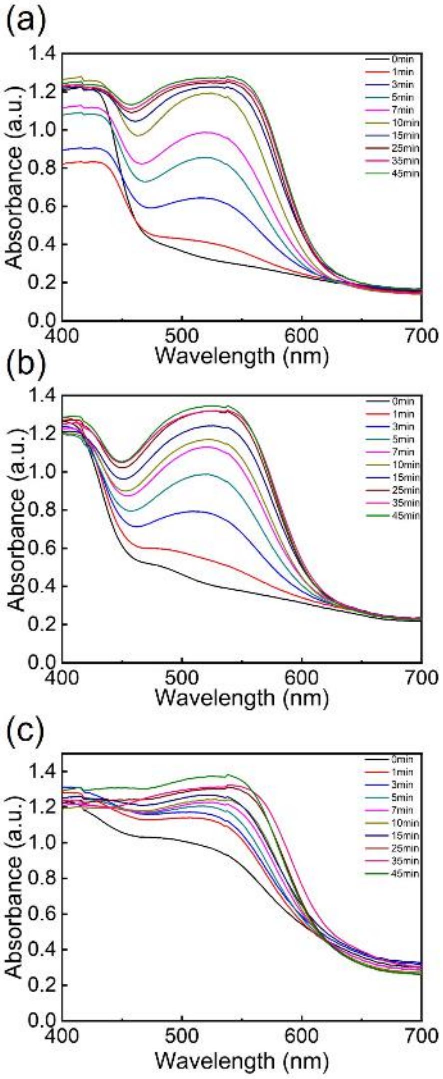

Figure S6. Recovery behavior of hydrogel composites with the different amounts of MBA (a: 5, b: 20, and c: 40 mg) after immersing in warm water thermo-stated at 50 °C.

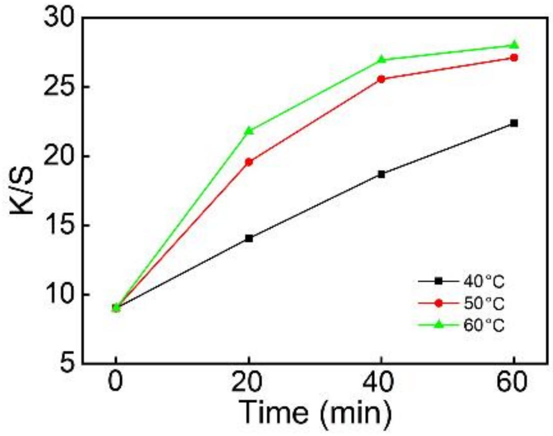

Figure S7. Effect of different recovery temperatures on the recovery process of the hydrogel composites with 5 mg of MBA: 40 ℃ (black), 50 ℃ (red), and 60 ℃ (green).

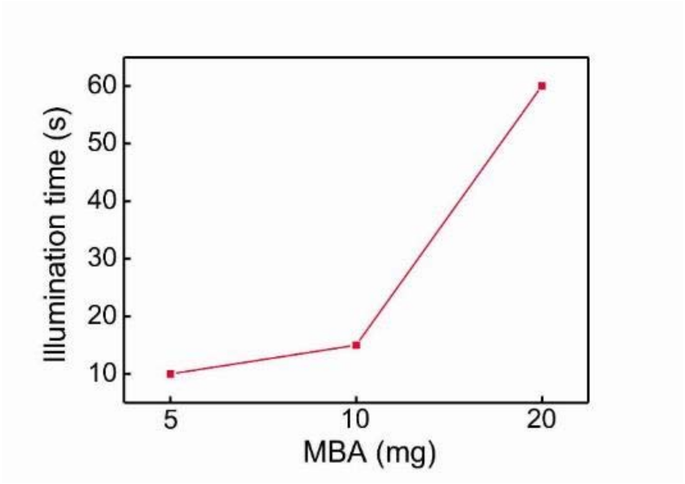

Figure S8. Illumination time required for the hydrogel composites to reach photochromic equilibrium as a function of the MBA content. The light intensity applied is fixed as 637 W m^{-2}.

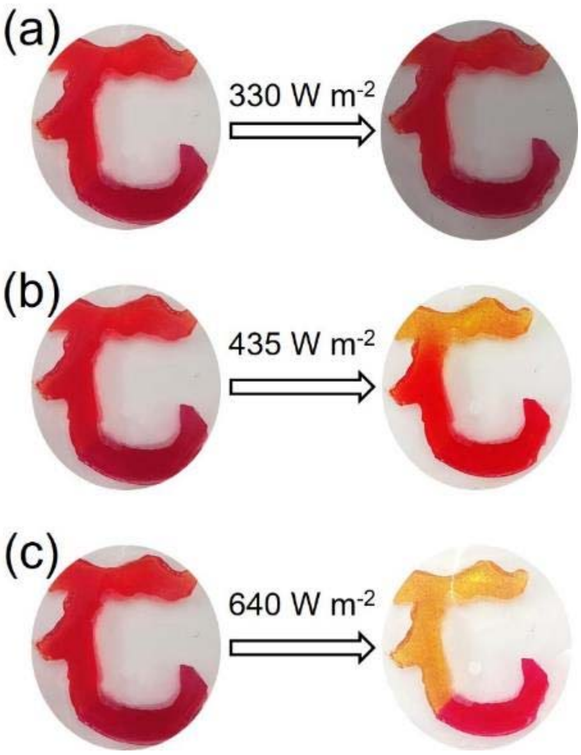

Figure S9. Discoloration of the hydrogel composites with different amounts of MBA (5, 20 and 40 mg) under natural light conditions (a: dark, 330 W m^{-2}; b: suitable, 435 W m^{-2}; c: bright, 640 W m^{-2}).

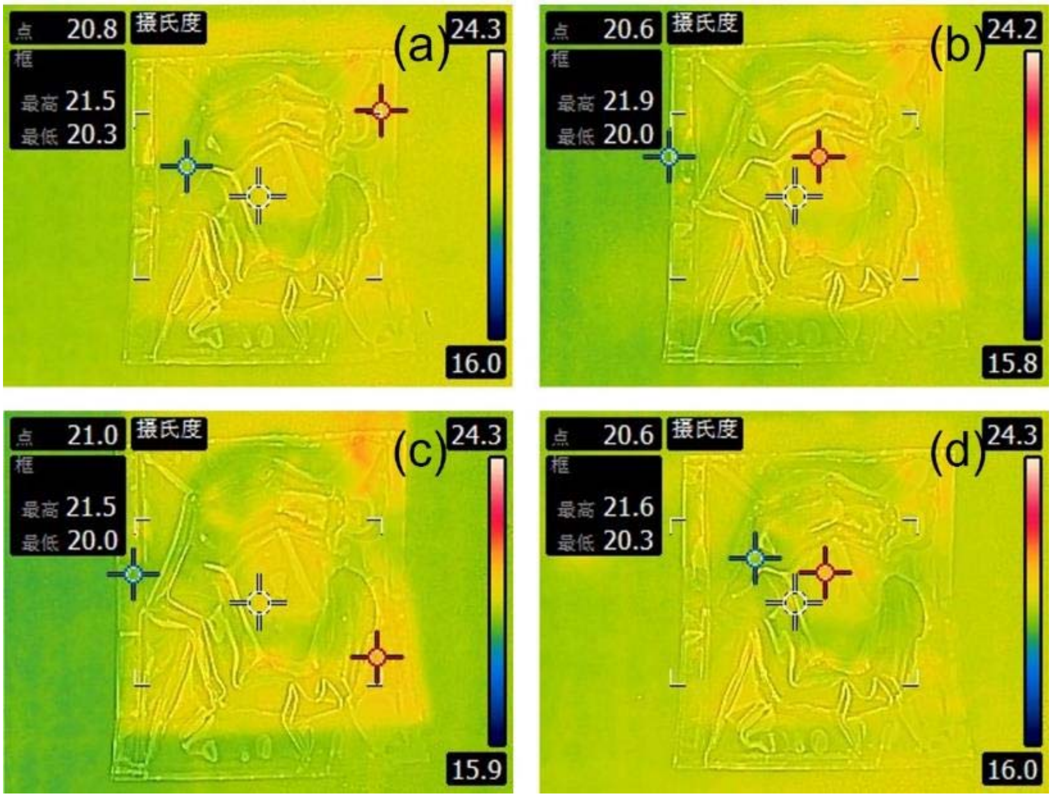

Figure S10. Temperatures of the hydrogel composite with 5 mg of MBA before and after illumination for different times (a: as-prepared, b: 10 s, c: 20 s, and d: 30 s). The light intensity applied is fixed as 637 W m^{-2}.
